# WaterHyacinth: A comprehensive image dataset of various Water hyacinth species from different regions of Bangladesh

**DOI:** 10.1016/j.dib.2023.109872

**Published:** 2023-11-29

**Authors:** Hasnain Kabir, Taslima Juthi, Md. Tarequl Islam, Md. Wahidur Rahman, Rahat Khan

**Affiliations:** a Department of Computer Science and Engineering, Khwaja Yunus Ali University, Sirajganj, Bangladesh; b Department of Computer Science and Engineering, Mawlana Bhashani Science and Technology University, Tangail, Bangladesh; c Department of Computer Science and Engineering, Uttara University, Dhaka, Bangladesh

**Keywords:** Eichhornia species classification, Computer vision, Deep learning, Image classification

## Abstract

The “WaterHyacinth” dataset, a recently gathered collection of images featuring four distinct species of Water hyacinth from different regions of Bangladesh, is presented in this article. There are four different classifications: *Lemna minor, Eichhornia crassipes, Monochoria korsakowii*, and *Pistia stratiotes*. The collection consists of 1790 original images and in addition 4050 augmented photos of Water hyacinth species. Every original picture was captured with the appropriate background and in sufficient natural light. Every image was correctly placed in its corresponding subfolder, providing optimal use of the pictures by various machine learning and deep learning models. Researchers could make major progress in agriculture, environmental monitoring, aquatic science, and remote sensing domains by utilizing this enormous dataset and various machine learning and deep learning approaches. In addition to opening opportunities for significant developments in these domains, it offers an essential asset for further study.

Specification Table

**Table:** 

Subject	Computer Sciences, Environmental Sciences, Agricultural Sciences.
Specific subject area	Identifying different species of Water hyacinth (Eichornia crassipes).
Data format	Raw, Analyzed, and Filtered.
Type of data	Image.
Data collection	Starting with a duration of three months, July 2023 to September 2023 we have collected raw images of total four species of Water hyacinth. We have collected these images from different locations of Sirajganj district and Pabna district. The images were taken using Redmi Note 8 Pro, Redmi Note 11, Samsung Galaxy S10, and iPhone 12 smartphones. The high-resolution photos were taken, and any low-resolution, noisy, or motion-blurred photos were eliminated from the dataset. Images of these four species have been captured in different light conditions, e.g., under direct sunlight, different day time such as in the morning, mid-day, afternoon, and under room light. We collected a total of 1,790 original images shared among the four species of Water hyacinth. Our dataset also gives 4,050 augmented images. In the whole process of data collection, we have tried to capture the images nearly natural as they look like in natural lights.
Data source location	1. Shahjadpur (6770), Sirajganj. 2. Enayetpur (6751), Sirajganj. 3. Sirajganj Sadar (6700), Sirajganj. 4. Kazipur (6710), Sirajganj. 5. Bera (6680), Pabna. City/Town/Region: Sirajganj and Pabna District, Rajshahi Division. Country: Bangladesh.
Data accessibility	Repository: Mendeley Data. DOI: 10.17632/vz6z64nwby.1 URL: https://data.mendeley.com/datasets/vz6z64nwby/1

## 1 Value of the Data


 

• The recommended Water hyacinth species database, known as the “WaterHyacinth” dataset, is useful because it has a vast collection of Water hyacinth species photos from four distinct plants that are commonly met in different parts of Bangladesh.
• “WaterHyacinth” an extensive dataset, contains 1,790 photos of Water hyacinth species that can be classified with human eyes. As a result, the researchers may effectively contribute to data analysis and categorize Water hyacinth categories.
• Various machine and deep learning-based approaches may be used to categorize [1], compare, test, and estimate data from the dataset.
• Furthermore, the dataset may be used to investigate other Water hyacinth use cases, such as animal and fish feed [2], compost and vermicompost production, biogas generation, and handmade paper [3], energy resource [4].
• The dataset has tremendous potential in environmental sciences, such as preserving nature, enabling Water hyacinth identification education, and emphasizing the importance of vegetation in the water.
• The “WaterHyacinth” dataset is accessible to the wider community, enabling academics to utilize it for their studies.

## 2 Objectives

The primary goal of generating the “WaterHyacinth” dataset is to classification different kinds of Water hyacinth [1], and which are good for environment and which are bad for environment. The dataset main motive is to remove water hyacinth that are bad for environment and use these for further industrial purposes such as handmade paper [3], cattle feed [2], organic beauty products etc. Water hyacinth is listed as the world's worst water weeds because it can cover approximately half a hectare in just 6–14 days [5]. Water hyacinth was assigned an effect value of 4 and a spread index of 3, showing its massive negative environmental impact. By lowering sunlight penetration, turbidity and dissolved oxygen, loss of nutrition, and disrupting the food-web, the weed diminishes species diversity [5]. The purpose of the dataset is to offer a wide-ranging collection of Water hyacinth images regarding to four species that are typically found in Bangladesh's diverse areas. These species consist of *Eichhornia crassipes, Lemna minor, Pistia stratiotes, Monochoria korsakowii*. This collection of data is intended to be used for building and evaluating machine learning and deep learning models for Water hyacinth identification [1], concentrating on the four species already mentioned. In addition, this data could be useful for the general population and educational initiatives, as well as sectors such as satellite imagery, agriculture, and the monitoring of the environment [6,7].

## 3 Data Description

The specified dataset “WaterHyacinth” includes a set of photos gathered from various rivers, ponds and swamps situated in Sirajganj, and Pabna district Bangladesh. The dataset contains 1790 original images and 4050 augmented images. These images are arranged in an organized folder. The primary folder is called “WaterHyacinth” and it contains two sub folders. One is original images that is our collected raw image files and other is augmented images. Both folders have four subfolders that is our four different species of Water hyacinth.

The names of the four Water hyacinth species listed below included in the “WaterHayacinth” dataset, associated with a short discussion:

**Water Hyacinth *(Eichhornia crassipes)****: Eichhornia crassipes* is commonly known as Water hyacinth*. Eichhornia crassipes* is a free-floating perennial aquatic plant. Its original habitat is South America [4]. It has thick, glossy, and ovate leaves that may extend up to 1 meter over the water’s surface. From its stem emerges a long, fibrous, multidimensional root, which is purplish black in color. A peduncle produces 8–15 attractive 6-petalled flower clusters. However, Water hyacinth is also considered one of the world’s most widespread aquatic weeds due to its ability to reproduce rapidly and produce thick mats on the surface of bodies of water involving lakes, rivers, and ponds. These thick mats can block sunlight, deplete oxygen levels in the water, and disrupt the natural ecosystem, harming native aquatic plants and animals. Water hyacinth is a plant with many advantages firstly because it can be used for many purposes like water treatments, medicinal uses, edible uses. We provide 450 original images in this category.

**Common Duckweed *(Lemna minor)*:** Duckweed a group of free-floating aquatic angiosperm plants of the family Lemnaceae classified as Monocotyledons. Duckweeds became the name because they are consumed by both fish and waterfowl. Because it eliminates excessive levels of nitrogen and phosphorus from rivers, common duckweed is utilized to mitigate the consequences of agricultural overflow. Duckweed has its beneficial side also [8]. Under ideal conditions its biomass gets doubled in 24 h. Duckweed acts as an excellent biofilter to treat domestic waste water. Fresh biomass of duckweed produced via domestic wastewater treatment plant can be integrated with fish and livestock production. This category contains 390 original images in this dataset.

**Water Lettuce *(Pistia stratiotes)*:** Water lettuce, scientifically known as *Pistia stratiotes*, is a free-floating aquatic plant characterized by its distinctive rosette of velvety, light green leaves. It is an aquatic plant that is native to Africa but has shown up in all tropical and subtropical waterways. This plant is most usually found in freshwater habitats such as ponds, lakes, and slow-moving rivers, especially within tropical and subtropical areas. Water lettuce is known for its rapid growth and ability to cover the surface of water bodies, which can lead to issues with water quality and ecosystem balance [9]. It can be used in home aquariums but should not be introduced into natural bodies of water. It can be used for many purposes like removal of chlorpyrifos [10], bioremediation, livestock feed, composting, biogas production. There are 480 original photos in this category within this dataset.

**Heartleaf False Pickerelweed (*Monochoria korsakowii):****Monochoria korsakowii* is a toxic herbaceous semi-submerged plant of the Pontederiaceae family that is found in paddy fields everywhere in the world*.* [11]. It is widespread in rice paddies and other bodies containing water, for a reason it is a threat for paddy field, and for soil. It is an annual or periodic herb that grows from a tiny root in water. The glossy green leaves grow up to 12 centimeters long and 10 cm broad on inflexible, hollow petioles. The inflorescence possesses 3–25 blossoms that open underwater and all at once. Each flower features six purple–blue tepals that are about a centimeter long. This plant also has commercial value since it is used as a forage crop [12] for livestock and poultry. A total of 450 original photos are in this category within this dataset.

## 4 Experimental Design, Materials and Methods

The evolution of the “WaterHyacinth” dataset there are six stages. The steps are image acquisition, image preprocessing, image augmentation, image compression and image classification. Each of these steps is briefly explained in this section.

### 4.1 Image acquisition system

There are four different classes of Water hyacinth in the dataset. The unprocessed leaf photographs were taken using several smartphones such as, redmi note 11 (50 MP, f/1.8, 26 mm (wide), 1/2.76″, 0.64 µm, PDAF), redmi note 8 pro (64 MP, f/1.9, 26 mm (wide), 1/1.72″, 0.8 µm, PDAF), iPhone 12 (12 MP, f/1.6, 26 mm (wide), 1.4 µm, dual pixel PDAF, OIS) from several locations in Bangladesh, including Shahajadpur, Enayetpur, Kazipur, Sirajganj and Pabna. All of the photographs were captured against a background of natural daylight. We used diffused lighting to highlight leaf details and reduce shadows when taking the images. Throughout the capture, we also kept the background a constant, natural one. Throughout the acquisition procedure, we made sure to check the image quality frequently. To account for differences in leaves, this required checking the focus, lighting, and taking photographs from different perspectives. We conducted quality control checks after gathering all the photographs. Images that had too much brightness, dim contrast, or blurry sections were found and removed from the collection of the dataset. This specific action was crucial in ensuring that only high-quality photos were included for later examination. We have ensured the dataset's integrity and improved the accuracy of subsequent analysis by removing these subpar photos. A subset of 1790 photos was selected from an initial collection of 2150 four different species photographs to create the dataset that was recommended for this study.

### 4.2 Image preprocessing

Initially we took total 2150 images of different Water hyacinth species images. Our images are in different dimensions as we took the images using different smartphones. After collecting the images, we manually deleted pictures that are in poor quality, have motion blur, noisy images, including images that have inappropriate backgrounds etc. After image preprocessing our dataset has total number of 1790 original images remaining.

### 4.3 Image partitioning

After image preprocessing, we have total 1790 images in our original dataset. Then we make four different folders in our original dataset. We renamed each folder with four Water hyacinth species names along with their scientific names. After that, we divided all the images and put them in the folders according to their species.

### 4.4 Image augmentation

The inclusion of variety in images through image augmentation increases the ability to make inferences and effectiveness of machine learning and deep learning-based classification models. We used the keras ImageDataGenerator module to increase the number of photos. A variety of picture augmentation techniques were used on the original dataset, including a random rotation, random noise, horizontal flips. Prior to splitting and placing the photographs in their specific class of subfolders for researchers to organize their studies according to their needs. All of the collected original photos went through image augmentation.

### 4.5 Image compression

**Fig. 1:**
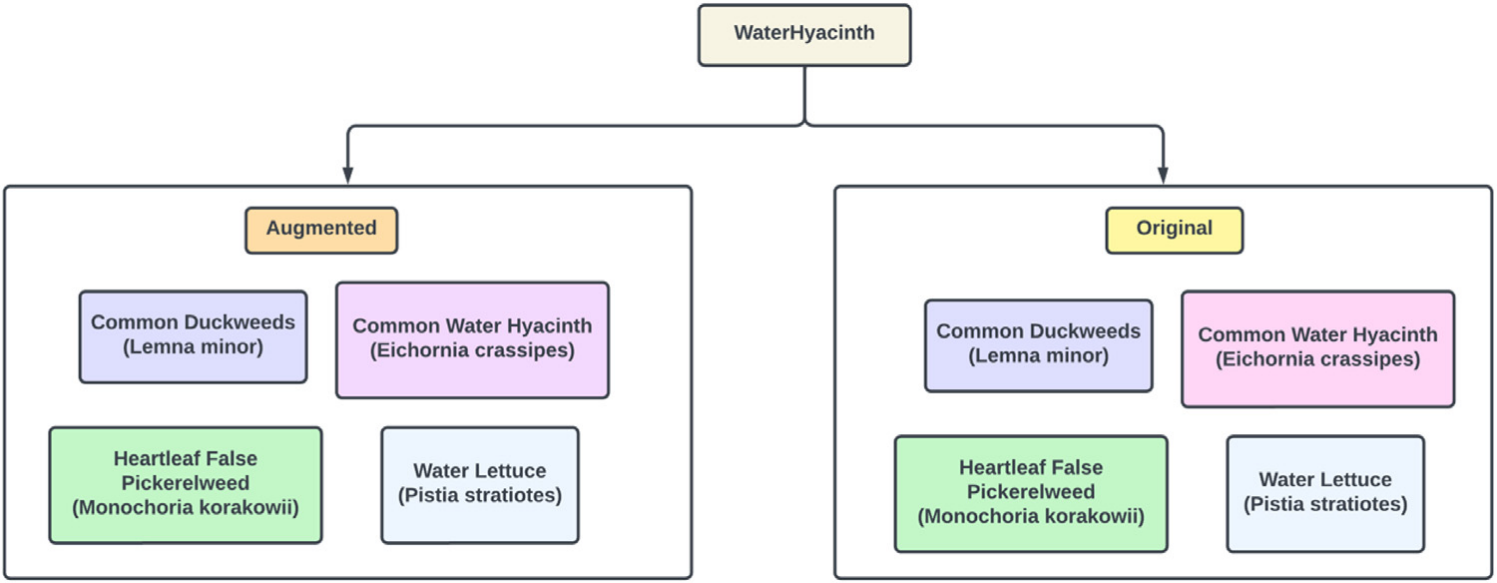
Data directory of WayerHyacinth dataset.

**Fig. 2:**
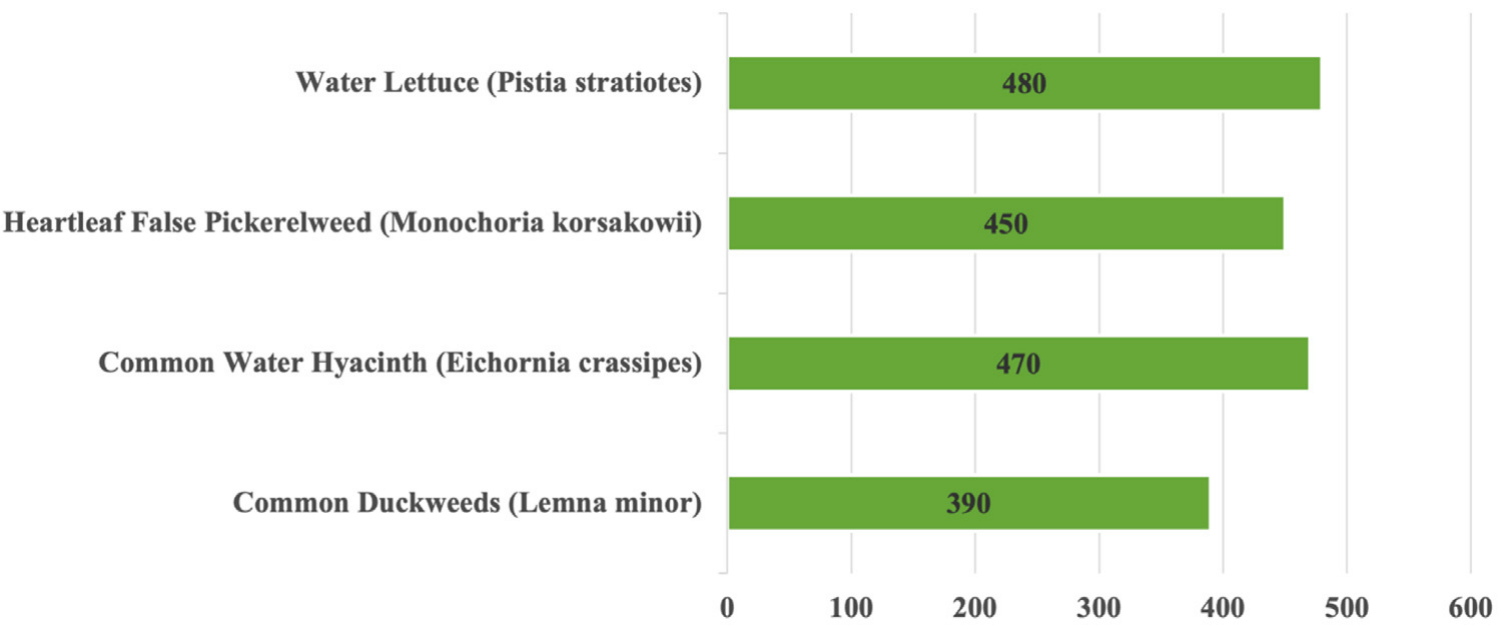
Water hyacinth species wise image distribution in WaterHyacinth dataset.

**Fig. 3:**
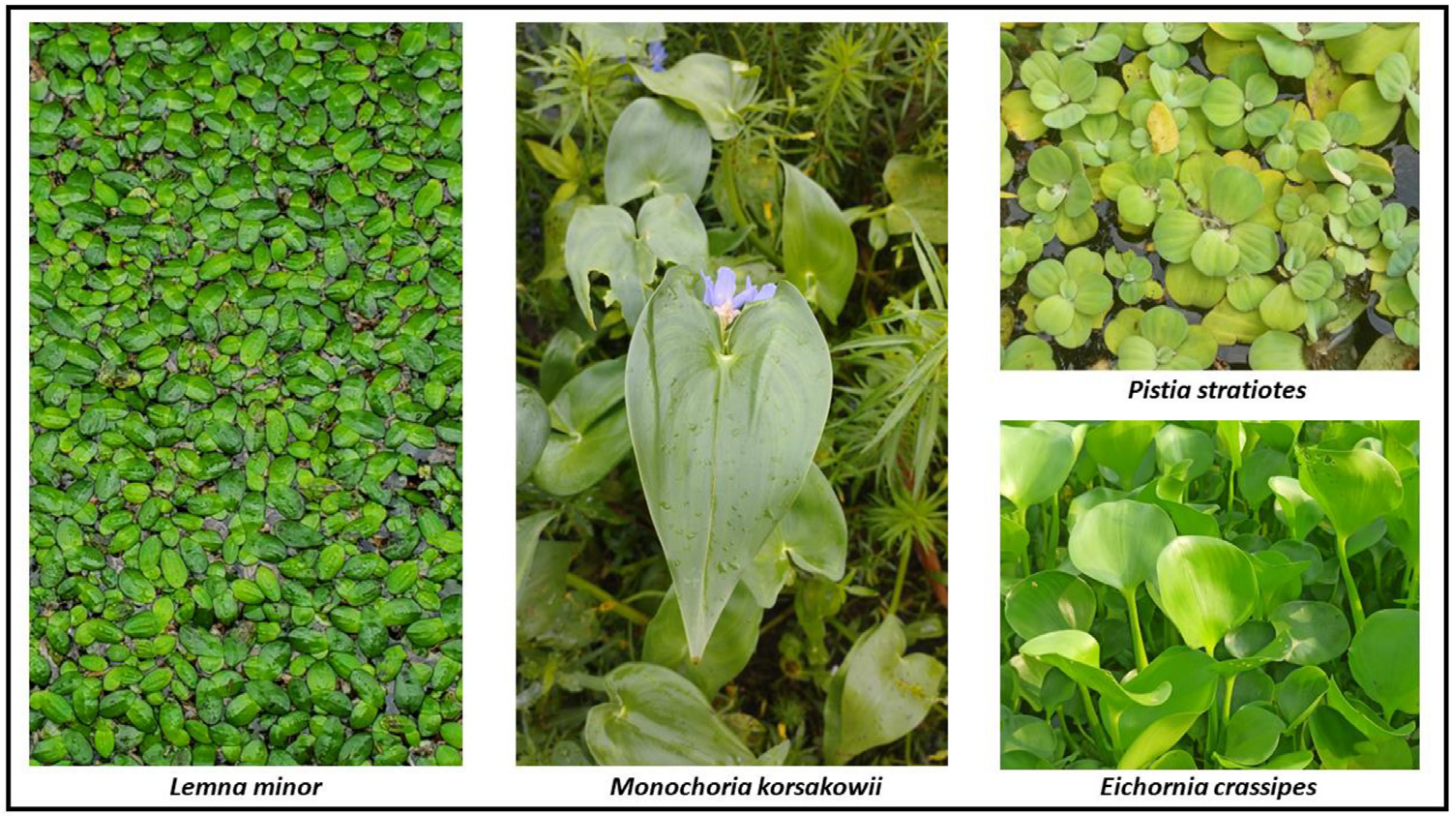
Some examples of images of each class in the WaterHyacinth dataset.

**Fig. 4:**
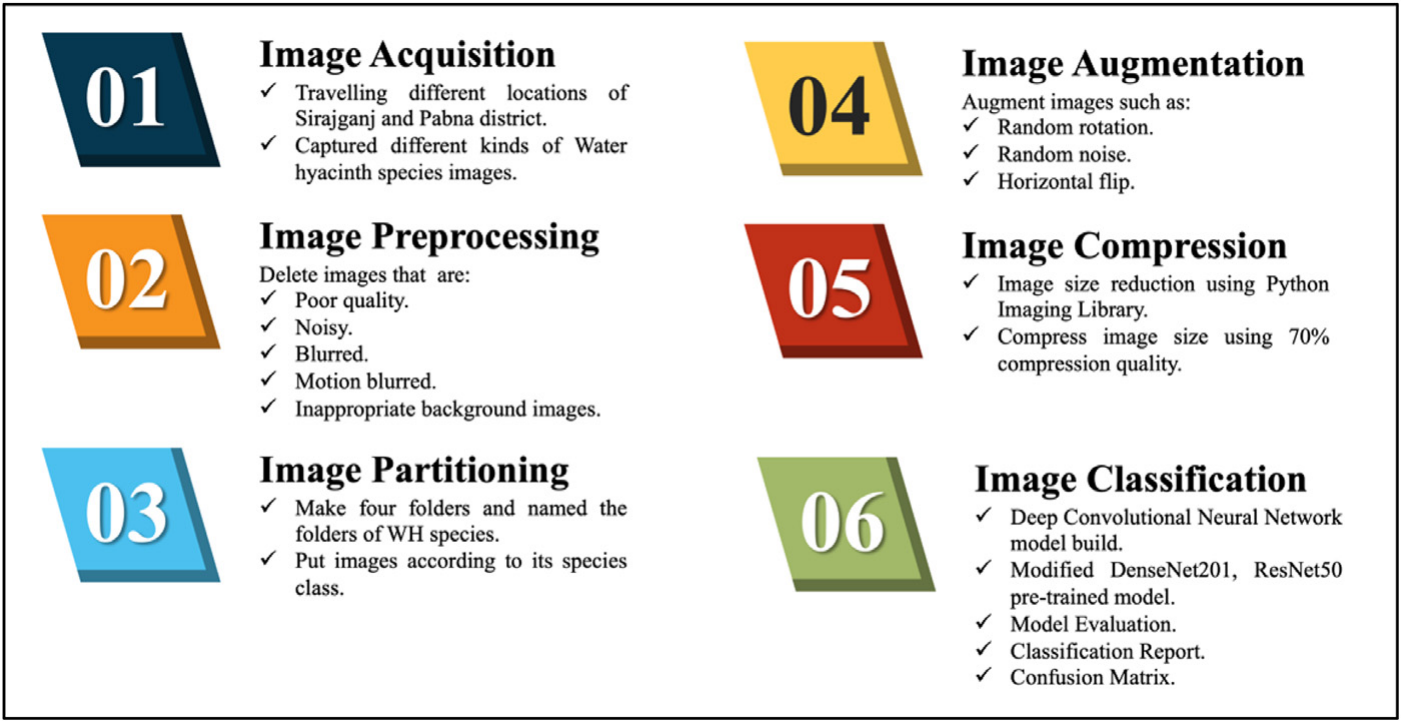
Development steps of WaterHyacinth dataset.

We compress image to minimizes the storage and transmission requirements by eliminating redundancy and irrelevant details in an image. It can be categorized into lossless compression, which retains all image details, and lossy compression, which sacrifices some details to achieve higher compression ratios. Our original image folder size was 4.29 GB and augmented image folder size was 15.7 GB and total dataset folder size was 19.99 GB. We need to upload our dataset in mendeley data. The available storage in mendeley data is 10 GB. So, we have to compress our dataset in this 10 GB available space. That's why we use image compression method to compress our images so that the dataset can be fitted in that 10 GB available storage. There is python library called Python Imaging Library (PIL) which offers variety of methods to work with images. In PIL library there is a method called image compression. We use that method for both of our original and augmented image folders and use compression quality to 70% which significantly reduces our image size. After using this compression method size of the image has reduced but the image quality or the dimension remains unchanged. After compression the original image folder size is 1.74 GB and augmented image folder size is 7.46 GB and thus total dataset folder size has become 9.2 GB which meets the available storage for uploading into Mendeley data (Fig. 1, Fig. 2, Fig. 3, Fig. 4).

### 4.6 Water hyacinth species image classification

**Fig. 5:**
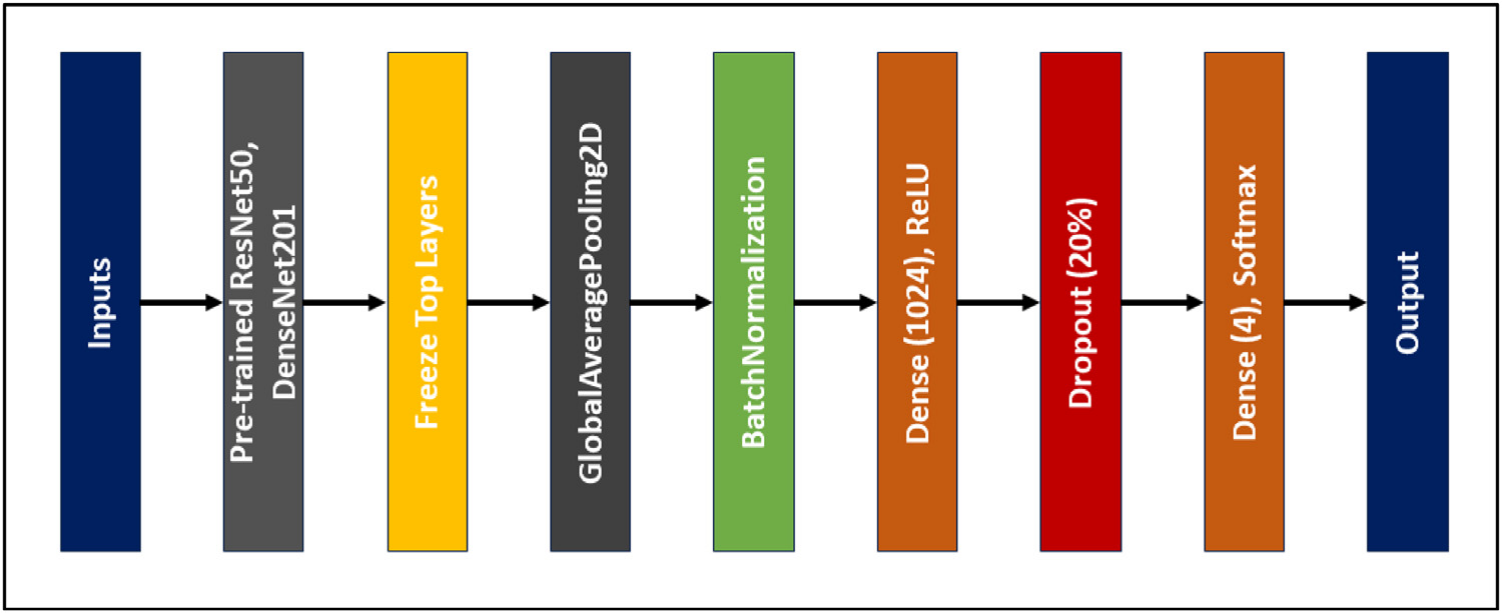
CNN model architecture that used For WaTerHyacinth evaluation.

For the assessment of the “WaterHyacinth” dataset we have used deep learning techniques. For this we choose convolutional neural network based deep learning models. We have chosen CNN because it can work well with image classifications. We evaluate the “WaterHyacinth” dataset with two well-known and lite pre-trained CNN models. These two models are ResNet50 [13], and DenseNet201 [14]. The pre-trained deep learning model known as ResNet50, or Residual Network with 50 layers, is often used for the classification of image applications. As a result of its 50 layers of neural networks, ResNet50 is distinguished by its depth, which enables it to recognize complex features and patterns in pictures. It was created specifically to deal with the issue of vanishing gradients, which can arise in extremely deep neural networks. Residual connections, often referred to as skip connections or shortcut connections, are used to accomplish this. ResNet50 is typically pre-trained on a huge dataset with millions of photos and thousands of classifications, like ImageNet. Another well-known pre-trained deep learning model is DenseNet201, which is short for densely connected convolutional networks with 201 layers. Image classification is its main use. It belongs to the family of convolutional neural networks called DenseNet. The densely linked structure of DenseNet201 enables deep feature propagation and feature reuse. Both the pre-trained models are intuitive to add into our own dataset classification issue because they are readily available in well-known deep-learning frameworks like TensorFlow and PyTorch. To train our model we just use our original dataset and split the dataset into three folders of seventy percent training dataset, twenty percent validation dataset and ten percent test dataset. This is achieved by a popular python library called python_splitter which can be very useful to split the dataset. After splitting the dataset every sub folder of train, validation and test contains the four sub folders of our species classes. Using another python module called ImageDataGenerator we preprocess our images before training the model. We resize every image of the dataset into (224 × 224) with 3 channels. It is because both of our pre-trained model accepts image shape of (224 × 224 × 3). The pre-trained weights from ImageNet remained intact for both models' construction, and both architectures’ top layers up until the final dense layer were frozen. Following the acquisition of every pre-trained element from each of the models, a fully connected network (FCN) with 1024 neurons was created by performing an operation of global average pooling and batch normalizing. To reduce the issue of overfitting in the models, ReLU activation was used in this dense layer and a dropout of 20% of neurons was implemented before the output layer. Due to the four distinct classes in our dataset, the final FCN layer only includes four neurons and softmax activation. As a result, there will be several classes in the categorization. Fig. 5 shows the pre-trained network architecture of both model which is used for WaterHyacinth dataset evaluation.

Following tables show the ResNet50 and DenseNet201 fully connected network architecture summary that are used to evaluate this dataset.

**Fig. 6:**
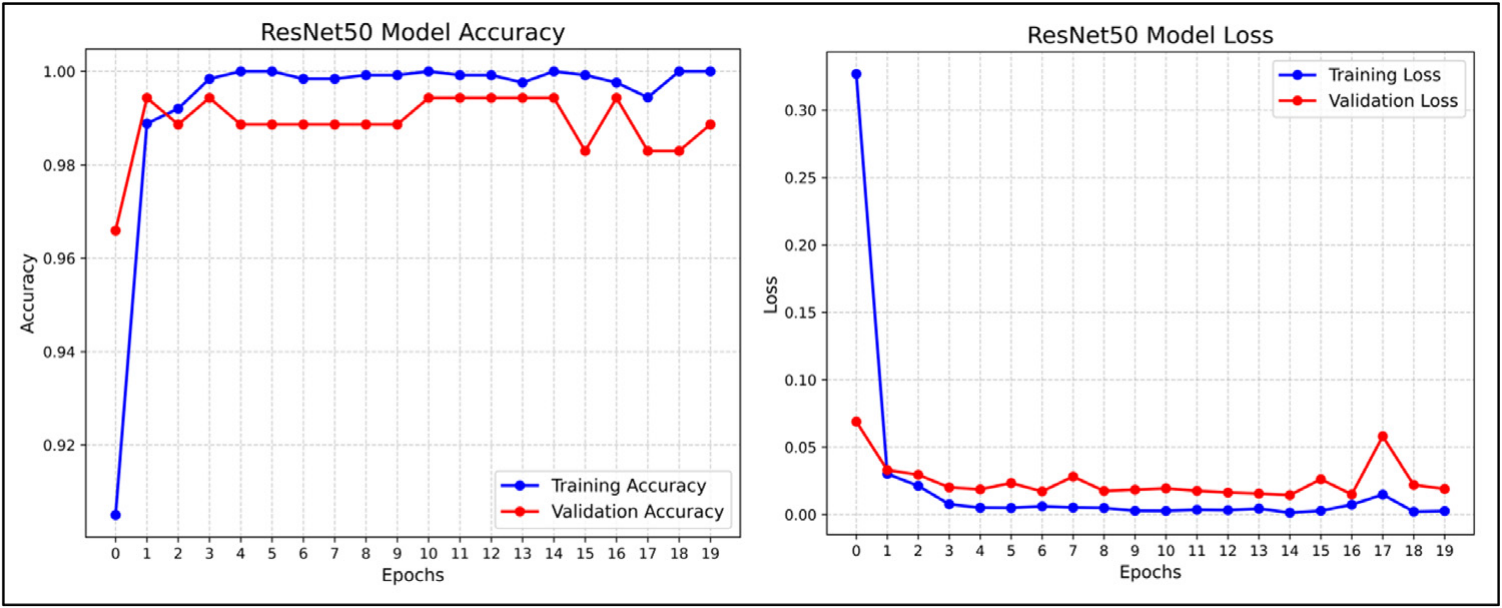
Training vs. Validation Accuracy and Loss For ResNet50 Model For The Proposed Dataset.

**Fig. 7:**
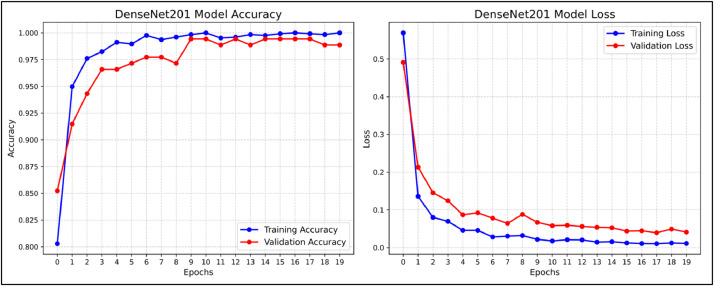
Training vs. Validation Accuracy and Loss For DenseNet201 Model For The Proposed Dataset.

To minimize the loss when training the model, an optimizer is utilized. We utilized the Adamax optimizer after creating the model and compiling it before initiating training with learning_rate set to 0.001. Adamax, a first-order gradient-based optimization technique, is a variation of Adam based on the infinity norm. It is appropriate for learning time-variant processes because it may modify the learning rate in response to data characteristics. After compile the model we trained our model for 20 epochs each. Following completing the model training we used plotting techniques to visualize our training and validation accuracy. The training vs. validation accuracy and loss of the ResNet50 model during 20 epochs is shown in Figs. 6 and 7 indicates training vs. validation accuracy and loss for the DenseNet201 models for 20 epochs. In the accuracy plot the X-axis represents the accuracy performance of training and validation data while the Y-axis represents number of epochs and in the loss plot X-axis represents the training and validation loss and Y-axis represents number of epochs during the training of both the models.

**Fig. 8:**
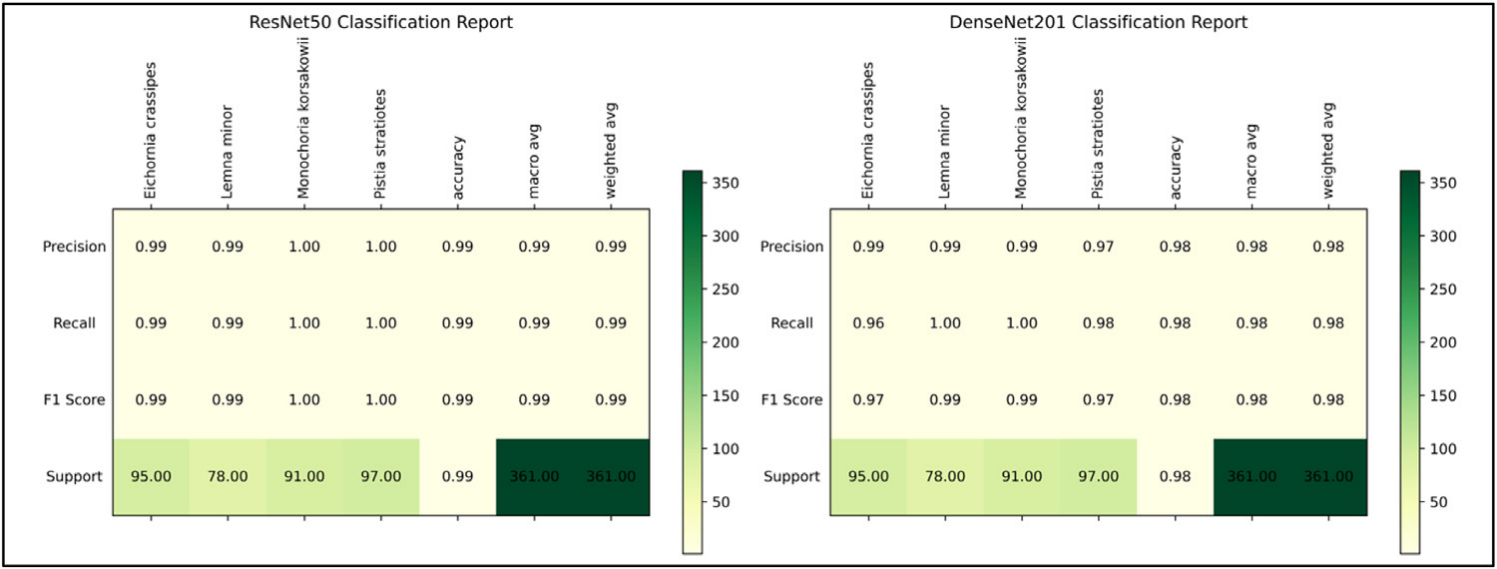
Classification Report of ResNet50 and DenseNet201 For The Proposed Dataset.

**Fig. 9:**
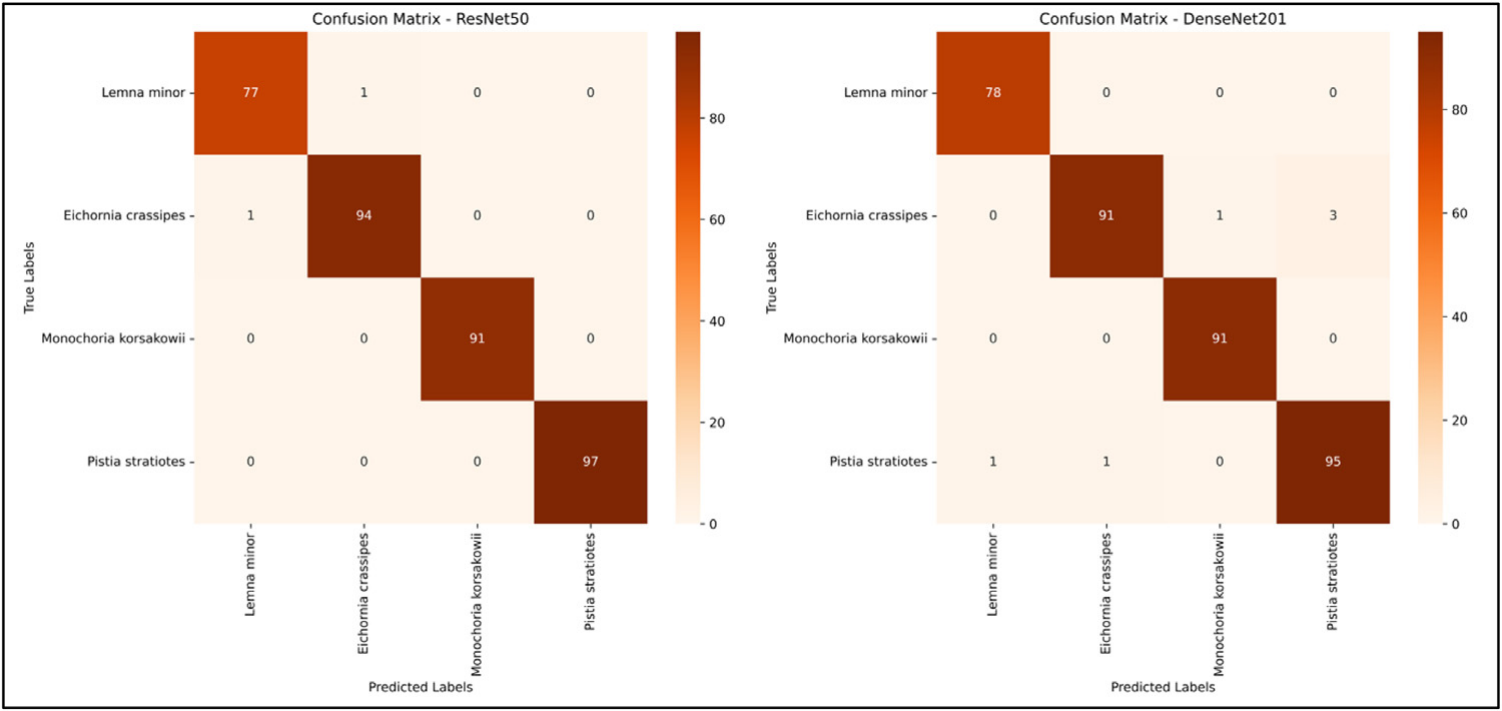
Confusion Matrix of ResNet50 and DenseNet201 For The Proposed Dataset.

We also evaluate the model with our test data and made a classification report and confusion matrix that shows our dataset species wise or can say class wise Precision, Recall and F-1 Score. Also, the confusion matrix provides to help with the visualization of how many much data from each class is correctly predicted and how much data is misclassified by both model. Fig. 8 shows the classification report of ResNet50-FCN and DenseNet201-FCN models where we can clearly see the Precision, Recall and F-1 score. Whereas Fig. 9 represents the confusion matrix of ResNet50-FCN and DenseNet201-FCN (Tables 1 and 2).

**Table 1:** Summary of the pre-trained DenseNet201-FCN architecture.

Layer	Shape	Number of parameters
Input layer	(224, 224, 3)	0
DenseNet201 (Functional)	(7, 7, 1920)	18321984
Global average pooling 2D	(None, 1920)	0
Batch normalization layer	(None, 1920)	7680
Dense layer (1024 units)	(None, 1024)	1967104
Dropout (20%)	(None, 1024)	0
Dense layer (4 units)	(None, 4)	4100

Total params: 20, 300, 868

Trainable params: 1, 975, 044

Non-trainable params: 18, 325, 824

**Table 2:** Pre-trained ResNet50-FCN architecture summary.

Layer	Shape	Number of parameters
Input layer	(224, 224, 3)	0
ResNet50 (Functional)	(7, 7, 2048)	23587712
Global average pooling 2D	(None, 2048)	0
Batch normalization layer	(None, 2048)	8192
Dense layer (1024 units)	(None, 1024)	2098176
Dropout (20%)	(None, 1024)	0
Dense layer (4 units)	(None, 4)	4100

Total params: 25, 698, 180

Trainable params: 2, 106, 372

Non-trainable params: 23, 591, 808

This outcome shows how well deep learning models were able to classify the species of Water hyacinth using the suggested “WaterHyacinth” dataset. When evaluating the pre-trained models’ performance, it was found that while both models’ training accuracy was identical, their validation accuracy varied. During the previous 10 epochs, the validation accuracy of the DenseNet201-FCN model has been fairly constant with the training accuracy. However, the validation accuracy of ResNet50 has varied significantly during model training. Yet, ResNet50-FCN outperforms DenseNet201-FCN somewhat when it comes to class predictions using test data. For the classes *Monochoria korsakowii* and *Pistia stratiotes*, the classification report in Fig. 8 compares the performance of ResNet50-FCN model with DenseNet201-FCN model. This is also reflected in the confusion matrix, where ResNet50-FCN categorized *Monochoria korsakowii* and *Pistia stratiotes* 100% appropriately, whereas for *Eichornia crassipes* and *Lemna minor* just one data was incorrectly classified as *Lemna minor* and *Eichornia crassipes*, respectively. *Eichornia crassipes*, however, has four incorrect data for DenseNet201-FCN, whereas *Pistia stratiotes* has two incorrect data. It is obvious that ResNet50-FCN is doing well in terms of classifying the suggested dataset. Table 3 shows the performance summary of ResNet50-FCN and DenseNet201-FCN model.

**Table 3:** Performance of the pre-trained ResNet50-FCN and DenseNet201-FCN for our proposed dataset.

Model	Train accuracy (%)	Validation Accuracy (%)	Test accuracy (%)
Pre-trained ResNet50-FCN (224 × 224 × 3)	100.00%	98.86%	99.45%
Pre-trained DenseNet201-FCN (224 × 224 × 3)	100.00%	98.86%	98.34%

## Limitations

Not relevant.

## Ethics Statement

The study was carried out carefully according to ethical guidelines, confirming a commitment to the highest standards. No water-dependent plants or aquatic animals suffered any damage throughout the data gathering process. The current study does not use any human beings, animals, plants, or data gathered from social media or other platforms, according to the authors, who have studied and abide by the ethical standards for publication through Data in Brief.

## CRediT authorship contribution statement

**Hasnain Kabir:** Data curation, Data curation, Formal analysis, Software, Conceptualization, Methodology, Investigation, Writing – original draft, Visualization. **Taslima Juthi:** Data curation, Data curation, Formal analysis, Writing – original draft, Resources, Conceptualization, Methodology, Software, Investigation. **Md. Tarequl Islam:** Conceptualization, Formal analysis, Project administration, Supervision, Writing – review & editing, Methodology, Resources. **Md. Wahidur Rahman:** Conceptualization, Formal analysis, Supervision, Project administration, Writing – review & editing, Software, Validation. **Rahat Khan:** Formal analysis, Conceptualization, Project administration, Supervision, Writing – review & editing.

## Data Availability

WaterHyacinth: A Comprehensive Image Dataset of Various Water Hyacinth Species From Different Regions of Bangladesh. (Original data) (Mendeley Data)
